# Überblick über die neuen EULAR-Empfehlungen zur antirheumatischen Therapie bei Kinderwunsch, in der Schwangerschaft und Stillzeit

**DOI:** 10.1007/s00393-025-01727-8

**Published:** 2025-09-24

**Authors:** Yvette Meißner, Frauke Förger

**Affiliations:** 1 Programmbereich Epidemiologie und Versorgungsforschung, Deutsches Rheuma-Forschungszentrum Berlin, Charitéplatz 1, 10117 Berlin, Deutschland; 2 Institut für Sozialmedizin, Epidemiologie und Gesundheitsökonomie, Charité – Universitätsmedizin Berlin, Berlin, Deutschland; 3 Klinik für Rheumatologie, HOCH Kantonsspital St. Gallen, St. Gallen, Schweiz

## Abstract

Im April 2025 wurden Empfehlungen der European Alliance of Associations for Rheumatology (EULAR) zur Anwendung von Antirheumatika bei Kinderwunsch, in der Schwangerschaft und in der Stillzeit publiziert. Sie aktualisieren und ersetzen die bisherigen „Points to Consider“ der EULAR von 2016. Die Task Force formulierte 5 übergreifende Prinzipien und 12 Empfehlungen für den Einsatz von Antirheumatika vor und während der Schwangerschaft, in der Stillzeit und bei männlichen Patienten. Übergreifend wird auf die große Bedeutung der effektiven Kontrolle der Krankheitsaktivität in Schwangerschaft und Stillzeit zum Schutz von Mutter und Kind hingewiesen. Anders als in der Version von 2016 werden Biologika, v. a. Tumornekrosefaktorinhibitoren, als unbedenklich sowohl in der Schwangerschaft als auch in der Stillzeit eingeschätzt. Hingegen wird größere Zurückhaltung bei der Verwendung von nichtsteroidalen Antirheumatika und Glukokortikoiden, insbesondere in höheren Dosen und über längere Zeiträume, empfohlen. Neu aufgenommen wurden Empfehlungen für Antirheumatika bei Männern mit Kinderwunsch sowie zu Impfungen im Säuglingsalter nach intrauteriner Biologikaexposition. Dieses Hot Topic fasst die EULAR Empfehlungen für die deutsche Rheumatologie zusammen.

## Abstract (en)

In April 2025, the European Alliance of Associations for Rheumatology (EULAR) published recommendations on the use of antirheumatic drugs in the reproductive phase, during pregnancy and while breastfeeding. The previous EULAR Points to Consider from 2016 were updated and replaced. The task force formulated five overarching principles and twelve recommendations for the use of antirheumatic drugs before and during pregnancy, while breastfeeding and in male patients. The overarching principles emphasise the great importance of effective control of disease activity during pregnancy and breastfeeding to protect mother and child. In contrast to the 2016 version, biologics, especially tumour necrosis factor inhibitors, are considered safe both during pregnancy and breastfeeding. On the other hand, greater caution is recommended when using nonsteroidal anti-inflammatory drugs and glucocorticoids, especially in higher doses and over longer periods of time. New recommendations have been included for antirheumatic drugs and male fertility as well as for vaccinations in infants after intrauterine exposure to biologics. This “Hot Topic” summarises the EULAR recommendations for German rheumatologists.

Die Betreuung von Patient:innen mit entzündlich rheumatischen Erkrankungen (ERE) in den Phasen von Reproduktion/Kinderwunsch, Schwangerschaft und Stillzeit ist eine anspruchsvolle Aufgabe. Bei Frauen muss das potenzielle Risiko einer antientzündlichen Therapie gegen das Risiko einer aktiven Erkrankung und die hiermit verbundenen Risiken für Mutter und Kind abgewogen werden. Bei Männern gilt, die Sicherheit von Antirheumatika in Bezug auf die Fruchtbarkeit und die Gesundheit des gezeugten Kindes zu berücksichtigen. Im Jahr 2016 wurden erstmalig „Points to Consider“ der European Alliance of Associations for Rheumatology (EULAR) für den Einsatz von Antirheumatika vor und während der Schwangerschaft sowie in der Stillzeit publiziert [1]. Diese wurden nun aktualisiert [2], um den Entwicklungen in der Behandlung von ERE gerecht zu werden und neue Daten zur Sicherheit der antirheumatischen Therapien in Schwangerschaft und Stillzeit, bei Männern mit Kinderwunsch und in Bezug auf Fertilität einzubeziehen. Angesichts des Umfangs und der Qualität der inzwischen vorliegenden Daten wurden nunmehr „Recommendations“ abgegeben, was einer höheren Stufe der Evidenz entspricht. Die Empfehlungen wurden nach den Regeln der EULAR durch ein multidisziplinäres Team von 27 Personen, darunter Rheumatolog:innen, Ärzt:innen anderer Fachrichtungen, Teratolog:innen, Methodiker:innen und Forschungspartnerinnen erarbeitet. Als Grundlage der Empfehlungen dienten eine systematische Literaturrecherche und Metaanalyse [3]. In diesem Hot Topic fassen wir die Empfehlungen zusammen und stellen die wichtigsten Änderungen für die klinische Versorgung in Deutschland vor [2].

## EULAR-Empfehlungen

**Abb. 1:**
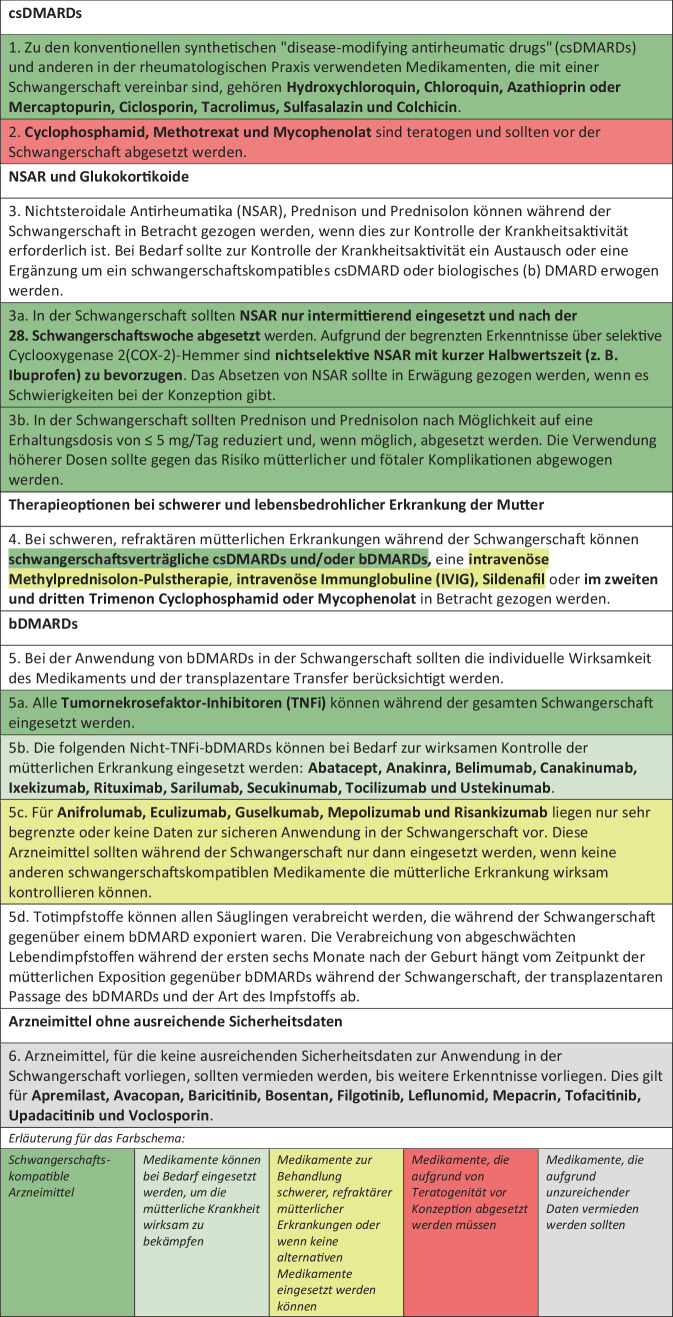
EULAR-Empfehlungen zu Arzneimitteln in der Schwangerschaft. Deutsche Übersetzung der EULAR-Empfehlungen [2]

Die Task Force formulierte 5 übergeordnete Prinzipien und 12 Empfehlungen, die in Tab. 1 und den Abb. 1, 2 und 3 in deutscher Übersetzung wiedergegeben sind.

**Tab. 1:** Übergeordnete Prinzipien. Deutsche Übersetzung der EULAR-Empfehlungen [2]

A) Allen Patient:innen sollte eine frühzeitige und regelmäßige Beratung im Hinblick auf Kinderwunsch und Schwangerschaft sowie zur Notwendigkeit einer Therapieanpassung angeboten werden
B) Die Behandlung von Patient:innen mit ERE vor der Konzeption und von Patientinnen während und nach der Schwangerschaft sollte auf eine Remission oder geringe Krankheitsaktivität abzielen
C) Das potenzielle Risiko einer Arzneimitteltherapie für den Fötus oder das Kind sollte gegen das Risiko einer unbehandelten mütterlichen Erkrankung abgewogen werden
D) Frauen sollten nicht vom Stillen abgehalten werden, wenn sie stillverträgliche Antirheumatika einnehmen
E) Die Wahl der Behandlung bei Kinderwunsch sowie während und nach der Schwangerschaft sollte ein gemeinsamer Entscheidungsprozess zwischen behandelnden Ärzt:innen und Patient:in sein

**Abb. 2:**
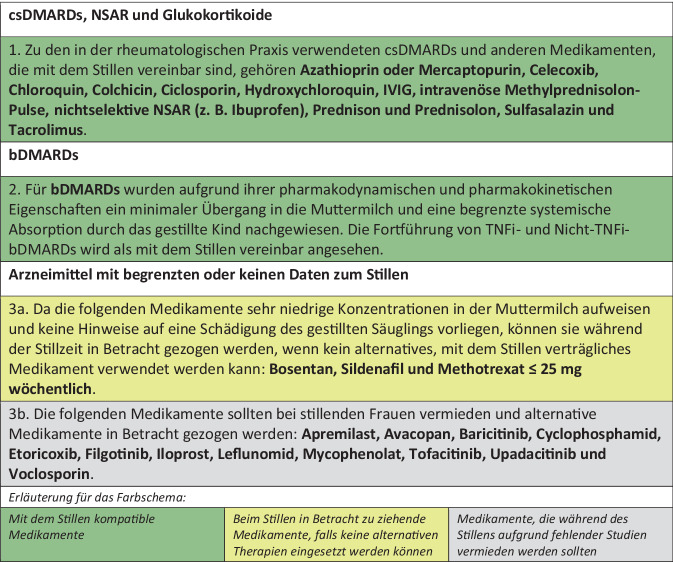
EULAR-Empfehlungen zu Arzneimitteln in der Stillzeit. Deutsche Übersetzung der EULAR-Empfehlungen [1]

**Abb. 3:**
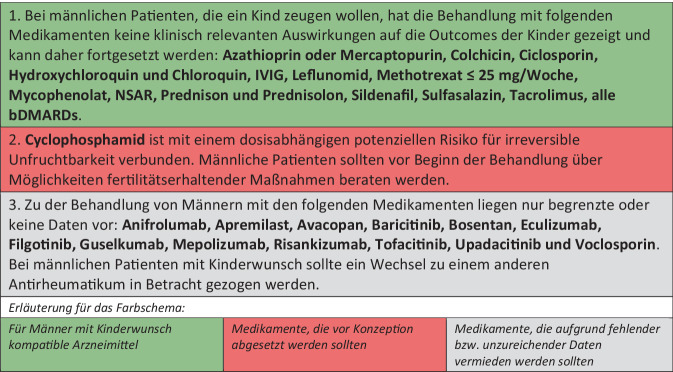
EULAR-Empfehlungen zu Arzneimitteln bei Männern mit Kinderwunsch. Deutsche Übersetzung der EULAR-Empfehlungen [1]

### Übergeordnete Prinzipien

Die übergeordneten Prinzipien geben die wichtigsten Aspekte für die Beratung von Patient:innen mit ERE und Kinderwunsch bzw. während Schwangerschaft und Stillzeit wieder. Es wird betont, allen Patient:innen frühzeitig und regelmäßig eine Beratung anzubieten, in der unter Einbeziehung der Fragen und Bedenken der Patient:innen auf die Bedeutung der Planung von Schwangerschaften und der Verwendung kompatibler Medikamente eingegangen werden sollte. Im Falle einer aktiven Erkrankung sollte die Schwangerschaft verschoben werden, bis eine optimale Krankheitskontrolle erreicht ist. Dies gilt auch für männliche Patienten, die planen, ein Kind zu zeugen. Eine unbehandelte mütterliche ERE ist mit einem relevant erhöhten Risiko für einen ungünstigen Schwangerschaftsverlauf und -ausgang verbunden. Neu ist Punkt D, der die Vorteile des Stillens unterstreicht. Demnach sollen Patientinnen über die positive Wirkung des Stillens bei der Einnahme von verträglichen Medikamenten informiert werden. Die Bedeutung einer gemeinsamen Entscheidungsfindung wird unter Punkt E hervorgehoben, um mögliche Sorgen zu adressieren und Patient:innen zu einer informierten Entscheidung befähigen zu können.

### Antirheumatika vor und während der Schwangerschaft

#### csDMARDs

Für Hydroxychloroquin, Chloroquin, Azathioprin oder Mercaptopurin, Ciclosporin, Tacrolimus, Sulfasalazin und Colchicin gibt es derzeit keine Hinweise auf eine erhöhte Rate an kongenitalen Fehlbildungen oder anderen Schwangerschaftskomplikationen. Hydroxychloroquin in Tagesdosen bis 400 mg sollte gegenüber Chloroquin bevorzugt werden. Bei Frauen mit normalem Thiopurin-Stoffwechsel kann Azathioprin in täglichen Dosen von bis zu 2 mg/kg Körpergewicht während der gesamten Schwangerschaft eingesetzt werden. Ciclosporin und Tacrolimus können während der Schwangerschaft in der niedrigsten wirksamen Dosis verwendet werden. Sulfasalazin kann in Dosen von bis zu 2 g/Tag während der gesamten Schwangerschaft eingesetzt werden. Da Sulfasalazin die Folatabsorption hemmt, wird eine gleichzeitige tägliche Folsäureergänzung empfohlen. Colchicin kann in Dosen von 1–2 mg/Tag eingesetzt werden.

Cyclophosphamid, Methotrexat und Mycophenolat sind nachweislich embryotoxisch und teratogen, d. h. eine Exposition während des ersten Trimenons der Schwangerschaft kann zu Fehlgeburten oder schweren Fehlbildungen führen. Teratogene Substanzen führen typischerweise zu einem charakteristischen Fehlbildungsmuster, wobei dies bei niedrig dosiertem Methotrexat nicht der Fall zu sein scheint. Patientinnen, die Cyclophosphamid, Methotrexat oder Mycophenolat erhalten, sollten eine wirksame Kontrazeption anwenden, und im Falle einer Familienplanung sollten die Medikamente vor der Konzeption abgesetzt werden (Cyclophosphamid: 3 Monate, Methotrexat: 1 bis 3 Monate, Mycophenolat: 1,5 Monate).

#### NSAR und Glukokortikoide

NSAR und Glukokortikoide können bei Bedarf zur Kontrolle einer aktiven Erkrankung in der Schwangerschaft verwendet werden. Jüngste Erkenntnisse zu Dosis- und Therapiedauer-abhängigen Effekten sprechen jedoch für einen zurückhaltenden Einsatz.

Es gibt keine Hinweise auf ein erhöhtes Risiko für Fehlgeburten oder Teratogenität bei Exposition gegenüber NSAR in der Frühschwangerschaft. Die beste Evidenz liegt für Ibuprofen vor, gefolgt von Diclofenac, während die Daten für COX-2-Hemmer begrenzt sind. Im zweiten Trimenon birgt eine kurzfristige Verwendung von NSAR (7 bis 10 Tage) keine wesentlichen Risiken für den Fötus. Unerwünschte Wirkungen auf das Ungeborene hängen in dieser Periode von der Expositionszeit, der Behandlungsdauer, der Dosierung und der Intensität der Prostaglandinhemmung ab. Daher empfiehlt die Task Force nichtselektive NSAR mit kurzer Halbwertszeit, z. B. Ibuprofen, in der niedrigsten wirksamen Dosis für kurze Zeit (7 bis 10 Tage). Nach der 28. Schwangerschaftswoche sollten NSAR abgesetzt werden, da in der Spätschwangerschaft NSAR-bedingte Risiken für den Fötus wie Oligohydramnion oder Verengung/Verschluss des fötalen Ductus arteriosus zunehmen. Da NSAR den Eisprung stören und die Fruchtbarkeit der Frau beeinträchtigen können, sollte bei Frauen mit Konzeptionsschwierigkeiten ein Absetzen erwogen werden.

Prednison und Prednisolon werden nicht mit einer erhöhten Rate schwerer Fehlbildungen in Verbindung gebracht. Zu den Risiken einer Glukokortikoidbehandlung während der Schwangerschaft über einen längeren Zeitraum oder in Dosen von mehr als 5 mg/Tag gehören jedoch schwangerschaftsbedingte Osteoporose, Schwangerschaftsdiabetes, schwere mütterliche Infektionen und Frühgeburten. Die Task Force empfiehlt daher eine restriktive Anwendung von oralen Glukokortikoiden bei Schwangeren.

#### bDMARDs

IgG-basierte Biologika durchlaufen den gleichen neonatalen Fc-Rezeptor(FcRn)-vermittelten transplazentaren Transfer wie natürliche mütterliche IgG-Antikörper, beginnend um die 20. Schwangerschaftswoche mit stetiger Zunahme bis zur Geburt. Die Bindungsaffinität an den plazentaren FcRn ist bei monoklonalen IgG1-Antikörpern (z. B. Adalimumab, Golimumab, Infliximab, Rituximab) am höchsten, bei Fc-Fusionsproteinen (Abatacept, Etanercept) gering und bei Fc-freien Molekülen (Certolizumab) unbedeutend. Dementsprechend können monoklonale IgG1-Antikörper zu neonatalen Medikamentenspiegeln führen, die die mütterlichen Spiegel übersteigen, was bei Fc-Fusionsproteinen und Fc-freien Molekülen nicht der Fall ist. Da eine In-utero-Exposition gegenüber bDMARDs in der zweiten Schwangerschaftshälfte die Planung einer Lebendimpfung beim Säugling beeinträchtigen kann, wird mit folgendem Ansatz sichergestellt, dass beim Neugeborenen keine oder nur minimale Arzneimittelspiegel auftreten:

- Absetzen aller IgG1-basierten Biologika bis Schwangerschaftswoche 20,
- Absetzen von Fc-Fusionsproteinen bis Schwangerschaftswoche 30 bis 32,
- Fortführung von Fc-freien Molekülen während der gesamten Schwangerschaft.

#### TNFi

Die Datenlage zur Sicherheit von TNFi in der Schwangerschaft hat sich seit 2016 deutlich verbessert. Die aktuelle Evidenz zeigt, dass TNFi nicht mit einem erhöhten Risiko für angeborene Fehlbildungen, Fehlgeburten oder andere Schwangerschaftskomplikationen verbunden sind. Auch ergab sich kein erhöhtes Risiko hinsichtlich schwerwiegender Infektionen bei Kindern innerhalb des ersten Lebensjahres nach einer intrauterinen Exposition gegenüber TNFi.

#### Nicht-TNFi-bDMARDs

Nicht-TNFi-bDMARDs scheinen die Rate unerwünschter Schwangerschaftsausgänge im Vergleich zur Allgemeinbevölkerung nicht zu erhöhen, jedoch ist die Evidenzlage schwächer als für TNFi. Die Therapie mit Belimumab oder Rituximab in der zweiten Schwangerschaftshälfte kann zu einer vorübergehenden B‑Zell-Depletion oder anderen Zytopenien beim Neugeborenen führen, jedoch ohne die Folge von schwerwiegenden Infektionen und mit Erholung der B‑Zellen innerhalb von 6 Monaten.

Obwohl keine Hinweise auf ein erhöhtes Risiko für Schwangerschaftskomplikationen vorliegen, sollten Anifrolumab, Eculizumab, Guselkumab, Mepolizumab und Risankizumab angesichts der sehr begrenzten Datenlage nur nach sorgfältiger Nutzen-Risiko-Abwägung verwendet werden.

#### tsDMARDs und weitere Arzneimittel mit unzureichender Datenlage

Apremilast, Avacopan, Baricitinib, Bosentan, Filgotinib, Leflunomid, Mepacrin, Tofacitinib, Upadacitinib und Voclosporin sollten wegen unzureichender Datenlage in der Schwangerschaft vermieden werden. Leflunomid sollte entweder 5 Halbwertszeiten (3,5 Monate) vor der Schwangerschaft abgesetzt oder es sollte ein Auswaschverfahren (z. B. mit Colestyramin) durchgeführt werden.

#### Therapie bei schweren und lebensbedrohlichen Erkrankungen der Mutter

Bei schweren, refraktären oder organ- bzw. lebensbedrohlichen Erkrankungen der Mutter während der Schwangerschaft sind die sichersten Behandlungsoptionen eine intravenöse Methylprednisolon-Pulstherapie, csDMARDs, bDMARDs, IVIG und Sildenafil, bei Bedarf auch in Kombination. Wenn es keine anderen Optionen gibt, können im 2. oder 3. Trimenon Cyclophosphamid oder Mycophenolat zur Behandlung von lebensbedrohlicher Erkrankung oder drohendem Organverlust gerechtfertigt sein.

#### Impfempfehlungen für Säuglinge nach bDMARD-Exposition

Der Impfplan für die ersten 12 Lebensmonate des Kindes kann die abgeschwächten Lebendimpfstoffe Bacillus Calmette-Guérin (BCG) und Rotavirus enthalten. Die BCG-Impfung sollte bei Säuglingen, die in utero TNFi-bDMARDs mit transplazentarem Transfer in der zweiten Schwangerschaftshälfte ausgesetzt waren (Adalimumab, Golimumab und Infliximab nach der 20. Schwangerschaftswoche, Etanercept nach der 32. Schwangerschaftswoche), um 6 Monate verschoben werden. Für Certolizumab gibt es einen minimalen bis keinen transplazentaren Transfer, und eine Therapie erfordert daher keine Änderung des Impfplans für das Kind. Die Rotavirus-Impfung kann bei Säuglingen, die in der Schwangerschaft gegenüber einem TNFi-bDMARD exponiert waren, nach dem üblichen Impfschema verabreicht werden.

Für Nicht-TNFi-bDMARDs gibt es insgesamt eine limitierte Datenlage zu Auswirkungen auf Lebendimpfungen bei in utero exponierten Säuglingen. Deshalb sollte bei Säuglingen, die im zweiten und dritten Trimenon gegenüber Nicht-TNFi-bDMARDs exponiert waren, Lebendimpfungen erst nach dem sechsten Lebensmonat erfolgen.

### Antirheumatika in der Stillzeit

#### Stillverträgliche Arzneimittel

Neu im Vergleich zu den „Points to Consider“ von 2016 ist, dass aufgrund der inzwischen vorliegenden umfangreichen Erkenntnisse alle bDMARDs als mit dem Stillen vereinbar angesehen werden.

Unverändert zu 2016 werden Azathioprin oder Mercaptopurin, Celecoxib, Chloroquin, Colchicin, Ciclosporin, Hydroxychloroquin, IVIG, intravenöse Methylprednisolon-Pulstherapie, nichtselektive NSAR (z. B. Ibuprofen), Prednison und Prednisolon, Sulfasalazin und Tacrolimus als mit dem Stillen vereinbar angesehen und sollten fortgesetzt werden. Bei intravenös verabreichter Methylprednisolon-Pulstherapie (1000 mg) sollte erst nach 2–4 h gestillt werden. Aufgrund der Sicherheitsdaten ist bei stillenden Müttern Ibuprofen gegenüber anderen NSAR zu bevorzugen.

#### Arzneimittel mit limitierter Evidenz für die Stillzeit

Die Task Force diskutierte intensiv die begrenzt verfügbaren Daten zu Bosentan, Sildenafil und Methotrexat ≤ 25 mg/Tag bei stillenden Frauen und kam zu dem Schluss, dass das Risiko während der Stillzeit akzeptierbar sei, wenn kein alternatives stillkompatibles Antirheumatikum verwendet werden kann. Denn die in die Muttermilch übergehenden Mengen dieser Medikamente sind sehr gering, und bei gestillten Säuglingen sind keine unerwünschten Ereignisse gemeldet worden.

Unzureichende Daten, jedoch nicht der Nachweis einer schädlichen Auswirkung auf die kindliche Gesundheit sind der Grund dafür, dass Apremilast, Avacopan, Baricitinib, Etoricoxib, Filgotinib, Iloprost, Leflunomid, Mycophenolat, Tofacitinib, Upadacitinib und Voclosporin bei stillenden Frauen vermieden werden sollten. Cyclophosphamid sollte ebenso wenig in der Stillzeit eingesetzt werden, denn es wurden 2 Fälle von Knochenmarksuppression bei Säuglingen beschrieben, die über die Muttermilch exponiert waren.

### Antirheumatika bei männlichen Patienten

#### Reproduktionsverträgliche Medikamente

Da eine hohe Krankheitsaktivität die männliche Fertilität beeinträchtigen kann, wird eine Krankheitskontrolle mit kompatiblen Medikamenten empfohlen. Es gibt derzeit keine Hinweise, dass Azathioprin oder Mercaptopurin, Colchicin, Ciclosporin, Hydroxychloroquin und Chloroquin, IVIG, Leflunomid, Methotrexat ≤ 25 mg/Woche, Mycophenolat, NSAR, Prednison und Prednisolon, Sildenafil, Sulfasalazin, Tacrolimus sowie alle bDMARDs negative Auswirkungen auf kindliche Outcomes wie Fehlbildungen haben. Dies gilt auch für Medikamente, die bei Frauen vor der Konzeption abgesetzt werden sollten, wie Methotrexat, Leflunomid und Mycophenolat. Bei Sulfasalazin ist ein reversibler negativer Einfluss auf die Spermienqualität nicht auszuschließen, hier könnte die Gabe von Antioxidanzien (z. B. Folsäure) von Vorteil sein.

#### Fruchtschädigende Medikamente

Cyclophosphamid ist mit einem dosisabhängigen potenziellen Risiko für irreversible Unfruchtbarkeit verbunden. Die Task Force empfiehlt, Cyclophosphamid mindestens 3 Monate vor dem Konzeptionsversuch abzusetzen und die Patienten zu Möglichkeiten fertilitätserhaltender Maßnahmen zu beraten.

#### Arzneimittel mit limitierter Evidenz

Zu der Behandlung von Männern mit Kinderwunsch und den folgenden Medikamenten liegen nur begrenzte oder keine Daten vor: Anifrolumab, Apremilast, Avacopan, Baricitinib, Bosentan, Eculizumab, Filgotinib, Guselkumab, Mepolizumab, Risankizumab, Tofacitinib, Upadacitinib und Voclosporin. Daher wird für diese Medikamente ein Wechsel zu einem anderen Antirheumatikum empfohlen.

Obwohl Studien keine negativen Auswirkungen von Filgotinib auf die Spermienqualität gezeigt haben, fehlen belastbare Daten zu Schwangerschafts-Outcomes nach väterlicher Exposition gegenüber Filgotinib und auch anderen Januskinaseinhibitoren. Daher empfiehlt die Task Force den Wechsel zu alternativen Substanzen, für die mehr Sicherheitsdaten vorliegen.

## Diskussion

Seit der Veröffentlichung der EULAR-„Points to Consider“ von 2016 ist das Wissen über die Arzneimittelsicherheit im Zusammenhang mit Familienplanung, Schwangerschaft und Stillzeit erheblich gewachsen, was zu der Aktualisierung und Hochstufung zu „Recommendations“ geführt hat. Alle Empfehlungen stützen sich neben einer systematischen Literaturübersicht und Metaanalyse auf einen Expertenkonsens mit einem hohen Maß an Übereinstimmung.

Im Vergleich zu 2016 gibt es wichtige Änderungen. Die Anwendung von Biologika wird großzügiger betrachtet. Unter den bDMARDs besitzen TNFi den höchsten Evidenzgrad hinsichtlich der Auswirkungen auf die Schwangerschaft und das Kind; sie sind auch am besten untersucht im Hinblick auf Stillzeit und männliche Fortpflanzung. Die Evidenz für Nicht-TNFi-bDMARDs ist begrenzter. Daher beruhen die Empfehlungen für Biologika mit begrenzten oder fehlenden Studiendaten auch auf Ähnlichkeiten der pharmakodynamischen und pharmakokinetischen Eigenschaften mit TNFi-bDMARDs. Es wird davon ausgegangen, dass komplett IgG-basierte Biologika ein vergleichbares Muster für den transplazentaren Transport in der zweiten Hälfte der Schwangerschaft und einen geringen bis minimalen Übergang in die Muttermilch aufweisen. Zudem wurde eine neue Empfehlung zur Impfung von Säuglingen mit In-utero-Exposition gegenüber bDMARDs aufgenommen. Bei stillenden Frauen gelten nun alle bDMARDs aufgrund ähnlicher Molekülgröße und pharmakokinetischer Eigenschaften als laktationsverträgliche Arzneimittel.

Weiterhin beinhalten die aktualisierten Empfehlungen eine restriktivere Anwendung von NSAR und oralen Glukokortikoiden bei Frauen vor und während der Schwangerschaft aufgrund potenzieller Risiken für Mutter und Kind, die mit Dosis und Behandlungsdauer zusammenhängen. Außerdem enthalten die Empfehlungen nun auch Hinweise zum Einsatz von Antirheumatika bei Männern mit Kinderwunsch.

Trotz der Fortschritte in den vergangenen 10 Jahren sind die Informationen über die Sicherheit einiger Antirheumatika in der Zeit der Reproduktion von Patient:innen mit ERE nach wie vor begrenzt. Um die relative Sicherheit von Medikamenten in der Schwangerschaft zu beurteilen, sind prospektive Beobachtungen und Vergleiche gegenüber nicht exponierten Patient:innen sowie gegenüber gesunden Kontrollen am aussagekräftigsten. Durch die enge Zusammenarbeit der europäischen Schwangerschaftsregister wird sich hier die Datenlage weiter verbessern [4, 5].

## Fazit für die Praxis

Zu den csDMARDs, die mit einer Schwangerschaft vereinbar sind, gehören Antimalariamittel, Azathioprin, Colchicin, Ciclosporin, Sulfasalazin und Tacrolimus. Bei NSAR und Glukokortikoiden wird ein restriktiverer Ansatz für die Anwendung in der Schwangerschaft empfohlen.

Auf der Grundlage einer individuellen Nutzen-Risiko-Bewertung können alle bDMARDs während der gesamten Schwangerschaft angewendet werden, vorzugsweise TNFi. Als stillkompatibel gelten alle bDMARDs sowie Antimalariamittel, Azathioprin, Colchicin, Ciclosporin, Glukokortikoide, IVIG, NSAR, Sulfasalazin und Tacrolimus.

Was die Verwendung von Arzneimitteln bei Männern betrifft, so sind Antimalariamittel, Azathioprin, Colchicin, Ciclosporin, IVIG, Leflunomid, Methotrexat, Mycophenolat, NSAR, Glukokortikoide, Sildenafil, Sulfasalazin, Tacrolimus und bDMARDs kompatibel, und die Therapie kann bei Patienten mit Kinderwunsch fortgesetzt werden.

Die aktualisierten Empfehlungen zu Antirheumatika in der Reproduktion, Schwangerschaft und Stillzeit sind geeignet, die Betreuung von Schwangeren mit rheumatischen Erkrankungen und ihren Kindern zu verbessern. Die Empfehlungen richten sich nicht nur an Rheumatolog:innen in der Versorgung, sondern auch an die nationalen Gesellschaften für Rheumatologie, Innere Medizin, Gynäkologie und Geburtshilfe, Familienmedizin, Pädiatrie, Pharmakologie, an nationale Informationsdienste für Teratologie sowie an Patient:innen. Es wird allen Kliniker:innen empfohlen, die Erkenntnisse in ihre tägliche klinische Praxis einzubeziehen.
